# Supplementary material for: Association between oral microbiome and seven types of cancers in East Asian population: a two-sample Mendelian randomization analysis
Source: Front Mol Biosci. 2023 Nov 21;10:1327893. doi: 10.3389/fmolb.2023.1327893 (PMC10702768; doi:10.3389/fmolb.2023.1327893)
Supplement: Supplementary file 1 [file DataSheet1.ZIP › Supplementary Materials/MR plots for tongue/tongue═╝/Gastric cancer/pheno.208_to_gastric cancer_forest.pdf]

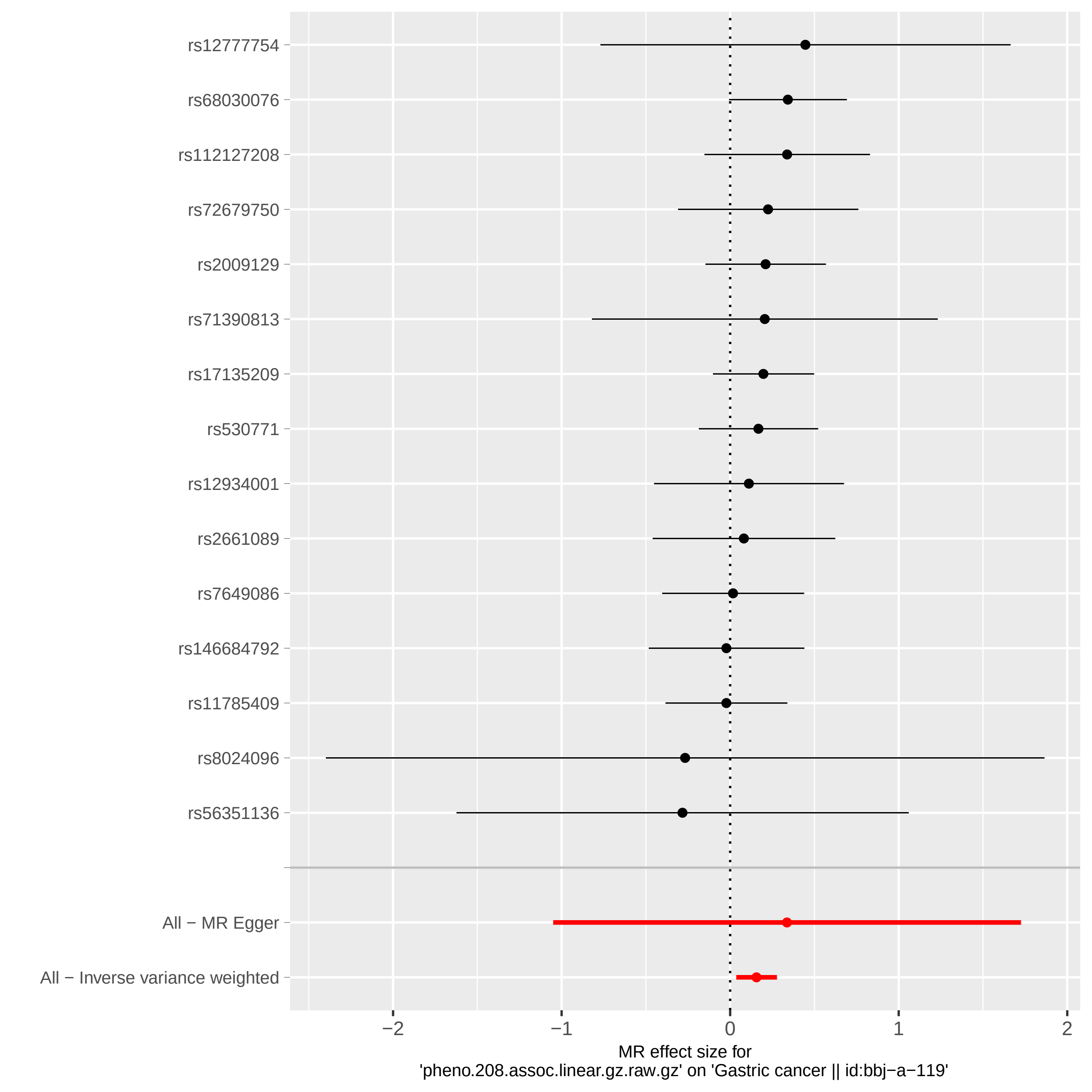
rs12777754
rs68030076
rs112127208
rs72679750
rs2009129
rs71390813
rs17135209
rs530771
rs12934001
rs2661089
rs7649086
rs146684792
rs11785409
rs8024096
rs56351136
All − MR Egger
All − Inverse variance weighted
−2
−1
0
1
2
MR effect size for
'pheno.208.assoc.linear.gz.raw.gz' on 'Gastric cancer || id:bbj−a−119'
